# Deep ensemble optimized models for probabilistic CTV breast segmentation

**DOI:** 10.3389/frai.2026.1777653

**Published:** 2026-04-30

**Authors:** Cecilia Riani, Maria Giulia Ubeira-Gabellini, Gabriele Palazzo, Giuseppe Ricciardi, Antonella del Vecchio, Alessandra Palma, Anna Balsamo, Angela Coniglio, Claudio Fiorino

**Affiliations:** 1 Medical Physics, IRCCS San Raffaele Scientific Institute, Milan, Italy; 2 Radiation Biophysics and Radiobiology Laboratory, Physics Department, University of Pavia, Pavia, Italy; 3 Centro Nazionale Intelligenza Artificiale e Tecnologie Innovative per la Salute, Istituto Superiore di Sanità, Rome, Italy; 4 Ministry of Health, Department of Human Health, Animal Health and Ecosystem (One Health) and International Relations (DOHRI), Rome, Italy

**Keywords:** breast cancer, breast radiotherapy, deep learning, model ensemble, probabilistic CTV, uncertainty

## Abstract

**Introduction:**

To optimize radiotherapy treatment and minimize toxicities, effective segmentation of organs-at-risk (OARs) and clinical target volume (CTV) is essential. Deep learning (DL) models can achieve high segmentation accuracy through careful tuning. However, their reliability also hinges on addressing uncertainties stemming from variability in clinical contouring practices. This study systematically evaluates six advanced DL models for automatic CTV segmentation in whole-breast radiotherapy. It further leverages the top-performing models to construct a probability map, aiming to improve consistency and mitigate bias in clinical predictions.

**Methods:**

Planning CTs from a single institute (861: training, 100: “temporal” test) were used to train six models for right/left breast CTV segmentation simultaneously: UNet, SegResNetDS, DynUNet (MONAI), nnU-Net (Total Segmentator), MedSAM2 (MS-A: CT-specific weights, MS-B: general medical-image weights). MedSAM2 employed a strong caveat: it needs a spatial prompt to make the prediction. Standard metric such as Dice Similarity Coefficient, Average Surface Distance and Hausdorff Distance were used to evaluate prediction compared to ground truth. A Friedman test followed by *post-hoc* pairwise comparisons through Conover test were conducted on the temporal test set. In addition, the best models derived (in line with inter-observer variability IOV) were used to build model-based probability maps and quantify the differences between high concordance (100%) and lower concordance (25%) isoprobabilities to clinician CTV.

**Results:**

All models attained overall satisfactory performance [comparable to IOV (Dice = 0.90)]. Among them, UNet, DynUNet, nnU-Net, and MedSAM2-A demonstrated the highest equivalent accuracy, with average ASD = 1.5 mm and HD95 = 3.8 mm. Conover test displayed more subtle differences with lower performance of two models out of six (SegResNetDS, MS-B), which were therefore removed for the subsequent built of a probability map. The analysis of the 100 vs. 25% isoprobability volumes on the temporal test dataset (average difference = 123 ± 81 cm^3^) highlighted areas of greater uncertainty at the lateral and cranio-caudal CTV borders.

**Discussion:**

Different DL architectures were optimized, trained and validated on a large cohort of patients, successfully predicting the CTV for both right/left breast. In addition, the first attempt to provide uncertainty maps was achieved through successful computation of DL model-based probability maps. Supported by CCM 2024 (Ministry of Health).

## 1 Introduction

Breast cancer (BC) is the most frequently diagnosed cancer in women worldwide with more than 2 million new cases in 2020 (Łukasiewicz et al., 2021). Its rising incidence and mortality are linked to evolving risk factors, improved cancer registration, and detection (Arnold et al., 2022). A standard treatment for early-stage BC includes breast-conserving surgery followed by adjuvant radiotherapy (RT), which reduces recurrence risk (Wang and Wu, 2023). Radiation therapy plan optimization is fundamental in the radiation therapy process, aiming to maximize therapeutic efficacy on clinical target volumes (CTV), while minimizing radiation induced damage to the surrounding healthy tissues, named organs at risks (OARs). This process relies on accurate 3D dose distributions computations, derived from the manual segmentations of target volumes and OARs. Segmentation is still typically performed manually. This is a highly time-consuming step, and it is prone to intra- and inter-observer variability (IOV) (Wang et al., 2019; Zhong et al., 2023). Deep learning (DL) models, if able to match the inherent limits of observer variability, may in principle significantly improve segmentation efficiency and precision, ultimately enhancing treatment planning and patient outcomes (Harrison et al., 2022).

Over the past years, a wide range of DL architectures have been proposed for medical image segmentation. The U-Net (Ronneberger et al., 2015) and its numerous variants (e.g., SegResNetDS, nnU-Net) have become the backbone of most segmentation pipelines. Large-scale frameworks such as nnU-Net [TotalSegmentator (Isensee et al., 2021)] and MedSAM2 (Ma et al., 2025) have further standardized training and benchmarking by providing highly adaptable pipelines across multiple tasks and anatomical regions. While this practice entered in clinical practice for OARs, it is still not yet used for CTV.

Despite progress in DL architecture, segmentation models still require large, high-quality annotated datasets, and external validation to assess models robustness. Comparative analyses on large cohorts are rare, and most works on breast CTV segmentation rely on 2D models rather than 3D ones (Men et al., 2018; Qi et al., 2022). Except for Ubeira-Gabellini et al. (2025), external validation studies are missing. In addition, to ensure reliability in RT, uncertainty quantification is crucial (van den Berg and Meliadò, 2022). Uncertainties may arise from errors and biases in the underlying datasets and CTV segmentations.

The CTV definition accounts for microscopic disease spread, which is not radiographically distinguishable from healthy tissue (Shusharina et al., 2020), therefore, its accurate quantification remains one of the biggest challenges in radiation therapy. According to ICRU Report (Jones, 1994), CTV is defined deterministically, forcing clinicians to make yes/no decisions regarding tumor delineation despite biological uncertainties/imaging limitations. As radiation oncology advances toward precision medicine, the need of improving CTV definition probabilistically, automating delineation through DL models, and creating models able to account for the biological response have become essential (Fiorino et al., 2020). Probabilistic concepts were first introduced for what regards coverage probability and dose-population histograms (Stroom et al., 1999; van Herk et al., 2000) to derive margin recipes, forming the basis for managing geometric uncertainties. These principles were extended (van Herk et al., 2003) by incorporating biological and fractionation effects of random geometric errors. Later, research moved toward eliminating rigid margins through probabilistic optimization, as in Bohoslavsky et al. (2013), which suggested to integrate uncertainty directly into planning optimization.

Therefore, the use of a probabilistic CTV, should ideally account for the different types of uncertainties such as: variations in patient positioning between fractions (Setup errors), intra-fraction and inter-fraction organ motion, tumor shrinkage/growth during treatment, limited resolution and/or poor contrast in imaging modalities, contouring IOV, biological uncertainty in microscopic disease spread, variability in radiosensitivity and tissue response (Unkelbach et al., 2018). This paradigm shift has led to increased focus on a systematic uncertainty characterization [macro typologies: epistemic and aleatoric uncertainty, well described in (Kendall and Gal 2017)], the introduction of Clinical Target Distribution (CTD) concepts and their future explicit incorporation in treatment plan optimization (Balvert, 2017; Unkelbach et al., 2018). Soon a new ICRU report focused on this (Jeraj, 2025) is expected. Recent studies have introduced alternative strategies to manage the fundamental CTV voxel-level uncertainty through alternative approaches like robust optimization and probabilistic definition of CTV (Balvert, 2017; Shusharina et al., 2018). In parallel, Brahme and Agren (1987) anticipated biologically driven planning by advocating non-uniform dose distributions for heterogeneous tumors. van Loon et al. (2012) advanced imaging-based prediction models for microscopic disease extension, reinforcing the biological dimension of uncertainty.

For what regards model uncertainty, not many studies have still been done, considering the relative recent introduction of DL. Epistemic uncertainty include Bayesian approaches like Bayesian Neural Networks (Blundell et al., 2015; Kwon et al., 2020; Gao et al., 2023) and its approximation Monte Carlo (MC) dropout (Gal and Ghahramani, 2016; McCrindle et al., 2021) or non-Bayesian methods as deep model ensembling (Lakshminarayanan et al., 2016; Mehrtash et al., 2020) and this error type can be reduced with more data (Erdur et al., 2024). Other suggested methods involving heatmap generation as the gradient-weighted class activation mappings (Grad-CAM) (Selvaraju et al., 2017). Aleatoric uncertainty, instead, include test-time augmentation (Wang et al., 2019) and learned loss attenuation (Kendall and Gal, 2017). Importantly, uncertainty evaluation directly relates to explainability by providing insights into AI algorithm's reliability and confidence levels. Clinical implementations include models such as probabilistic Unet, combining variational autoencoders with Bayesian inference (Kohl et al., 2019), MC dropout-based frameworks (Wickstrøm et al., 2020; Jungo et al., 2020; Maruccio et al., 2024) or attention mechanisms (De Biase et al., 2023; Biase et al., 2025).

**Figure 1:**
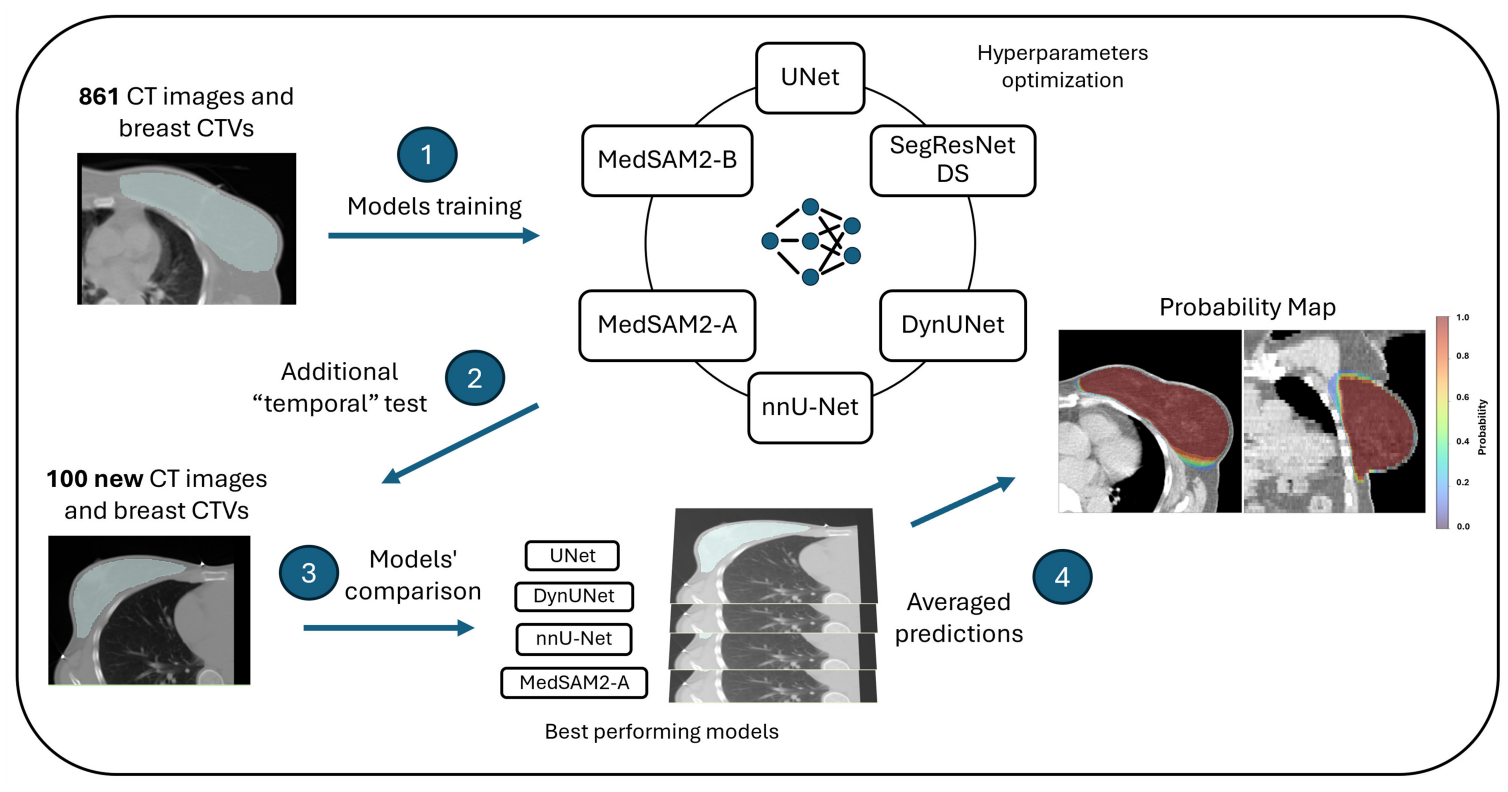
Graphical summary. (1) A large cohort of 861 images and breast Clinical Target Volumes of patients undergoing adjuvant radiotherapy were collected and used to train and validate six Deep Learning models (UNet, SegResNetDS, DynUNet, nnU-Net, MedSAM2-A, MedSAM2-B). (2) After training and validation, each model was also tested on an new cohort of 100 images corresponding to patients treated after the training cohort (“temporal” test). (3). Models predictions were compared through statistical test. (4). The four best performing models were used to derive a probabily map per each patient by averaging the four predictions obtained.

The approach of the current study consisted in the evaluation of multiple architectures and hyperparameters optimization (HPO), not done in previous breast CTV studies, crucial to enhance model's performance. We compared established frameworks such as UNet, SegResNetDS, DynUNet, nnU-Net and recently developed foundation models as MedSAM2 through standard segmentation metrics. We performed an internal test on an independent dataset of 100 new patients, enabling a more comprehensive comparison across different models (Figure 1). Furthermore, we built an ensemble-based probability map from the four best-performing models aiming to offer a reliable tool for clinical quality assurance for CTV breast segmentaion.

## 2 Materials and methods

### 2.1 Dataset

The dataset used for model comparison consisted of 3D planning CT images and CTV contours delineated by the treating clinicians, as detailed in Vincenzi et al. (2025); Fodor et al. (2022). In short, planning target volume (PTV) was generated by expanding the CTV with a 5–10 mm margin in all directions, except for the lung (5 mm); PTV was cropped to avoid the first 5 mm of skin. Data refer to 861 patients treated at the San Raffaele Institute, Milan, who underwent breast-conserving surgery followed by radiotherapy. All images were acquired with the same scanner (GE Medical System - HiSpeed NX/i). CTV delineation was performed by several clinicians in the period 2017–2021. In addition, for the specific aim of this work, another dataset, of 100 additional patients treated after 2021, was used to provide a “temporal” test of the best models. In Table 1 the number of labels produced by each clinician [A-P] are reported divided into training and test patients. For what regards the image pre/post-processing applied, we refer to our previous paper (Ubeira-Gabellini et al., 2025).

**Table 1:** Numerosity of labels for each clinician divided in train/val and test (temporal test).

Clinicians	A	B	C	D	E	F	G	H	I	L	M	N	O	P	Unknown	All
# Train/Val	88	9	70	118	21	19	73	14	290	18	63	59	1	0	18	861
# Test	13	0	1	1	2	1	11	0	27	0	16	24	0	1	3	100

The table reports the number of labels available per clinicians, considering all available data (the ones used for training and temporal test). All clinicians have labeled different images.

### 2.2 Hyperparameters optimization of three models

All models were trained on a Nvidia DGX01, using eight CPU cores (CPU: 2 × Intel(R) Xeonn(R) CPU E5- 2698 v4 @ 2.20GHz), 108 GB of RAM, and one Nvidia Tesla V100 SXM2 32 GB. Six 3D deep learning architectures were considered. The first three models, UNet, SegResNetDS, and DynUNet, are CNN-based models implemented in MONAI (v1.3). UNet extends the standard architecture (Ronneberger et al., 2015) by incorporating ResidualUnit blocks (He et al., 2015) and skip connections to mitigate vanishing-gradient issues. SegResNetDS retains an encoder–decoder structure but employs a deeper encoder and smaller decoder, and supports deep supervision, which enhances performance and accelerates convergence (Dou et al., 2016; Lee et al., 2014). DynUNet provides broader flexibility in handling diverse datasets (Isensee et al., 2018, 2021).

Since pretrained weights were unavailable, these three models underwent hyperparameter optimization (HPO) using Optuna (Akiba et al., 2019). The Study module handled the objective function, a TPE sampler explored the hyperparameter space, and a Median pruner terminated unpromising trials early. HPO was performed on 400 images (80% training, 20% validation) to allow more trials. All trials used DiceLoss, selecting configurations with the highest Dice Similarity Coefficient (Dice). Optimization details are reported in Table 2, and the resulting best hyperparameters were used for subsequent experiments. Additional loss functions (DiceCE, Tversky, HausdorffDT), also from MONAI, were evaluated to explore potential convergence improvements. Pre-processing and post-processing for UNet, SegResNetDS, and DynUNet followed the procedures detailed in Ubeira-Gabellini et al. (2025).

**Table 2:** Computational times for training different models on 861 images.

Model	UNet	SegResNetDS	DynUNet	nnU-Net	MS-A	MS-B
Computational time	16 h	36 h	49 h	5 h	5 h	13 h
Best epoch	198/200	198/200	198/200	197/200	200/200	499/500

### 2.3 Fine tuning of two state-of-the-art DL models

In addition, we considered two state-of-the-art DL models. The first is the nnU-Net framework (v2) in its “3D full-resolution” configuration, as employed in TotalSegmentator (Isensee et al., 2021). The second is the MedSAM2 architecture (Ma et al., 2025), initialized with distinct pre-trained weights (CT-Lesion and Latest). These architectures did not require HPO due to their built-in validation strategies. The same pre- and post- processing strategy applied to MONAI models was also applied to the nnU-Net predictions (see Appendix Figure A3): the images were cropped according to (Ubeira-Gabellini et al., 2025) and the model was trained for 200 epochs dividing the dataset in 80% for training and 20% for validation. For fine tuning MedSAM2, ground-truth masks were refined by removing scattered labels and discarding components smaller than 1,000 voxels in 3D using connected-component filtering. Images were windowed (level = −50, width = 300), normalized to [0, 255], cropped to non-zero slices, rescaled to 1,024 × 1,024 pixels, and converted to three-channel representations to match model input requirements. The resulting paired images and masks were used to train two models: one initialized from CT-Lesion weights called MedSAM2-A (MS-A) and one from Latest weights MedSAM2-B (MS-B), which are general medical-image weights. MedSAM2 needs a spatial prompt, that in our case was a bounding box a priori to make the prediction. During inference, volumes were clipped to [−300, 250], normalized, resized to 512 × 512, and converted to three-channel tensors. The z-extent was fixed by scanning the auxiliary mask to identify the first and last slices containing foreground, while the 2D bounding-box prompt was derived from the slice with the largest foreground area. Segmentation was propagated forward from the first slice and backward from the last slice, and the final prediction was defined as the intersection of the two propagations. This procedure constrained inference to a fixed cranial caudal span, improving z-axis consistency. Training of the nnU-Net and MedSAM2 architectures was performed using their default built-in loss functions, without any modification (see Appendix Figure A2).

### 2.4 Performance evaluation and statistical test

**Figure 2:**
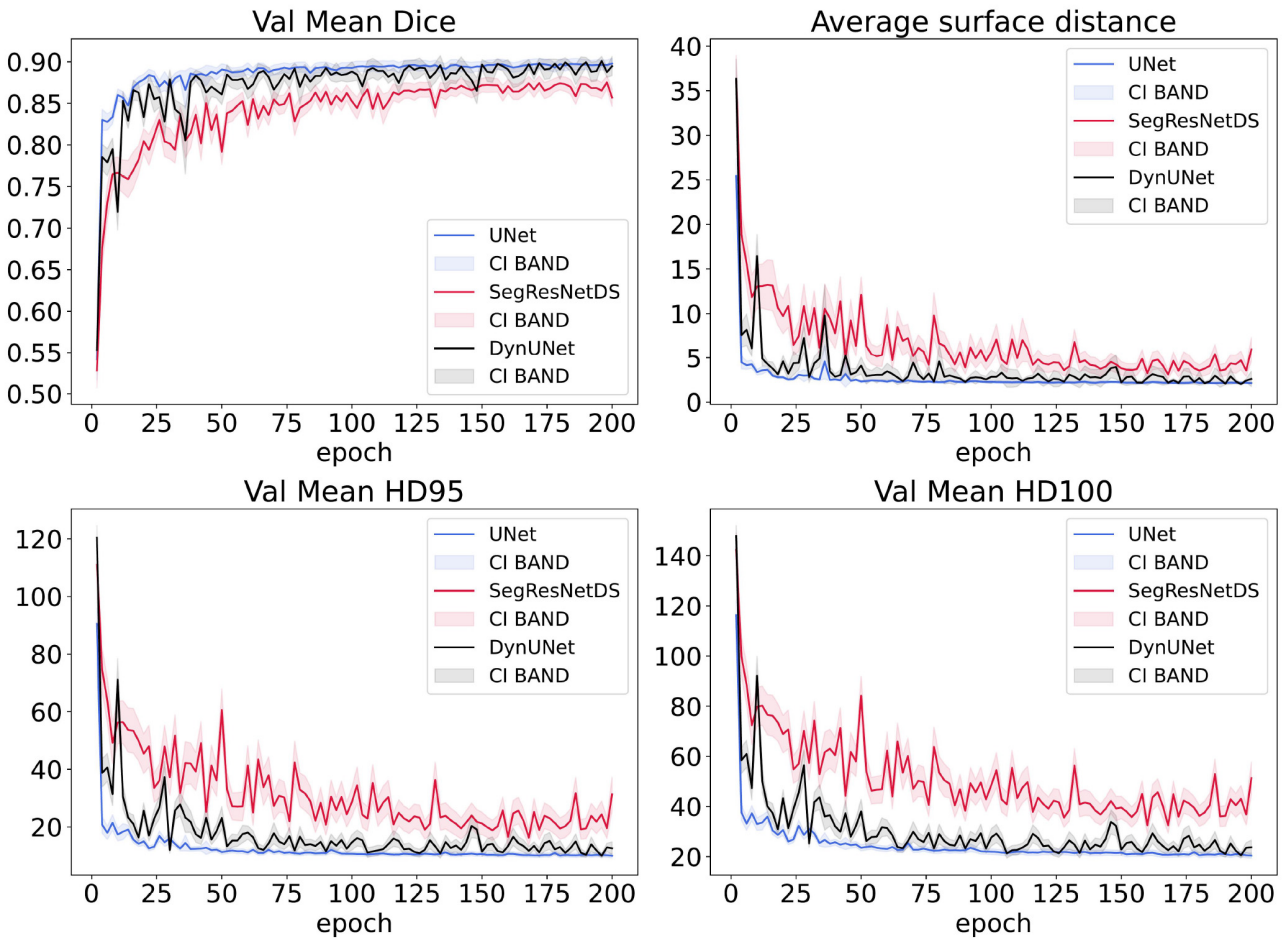
Loss/metrics comparison between segmentation models. Metrics over 200 epochs during the validation process for three models: Unet (blue), SegResNetDS (red), DynUnet (black). The training loss function used is DiceLoss for UNet and SegResNetDS and DiceCELoss for DynUNet. The corresponding bands represent the 95% confidence interval (CI) for each metric.

The segmentation performance of the validation dataset over training epochs, was addressed through Dice similarity coefficient (Dice), Jaccard Index (IoU), Symmetric Average Surface Distance (ASD), Hausdorff distance (HD) and its 95th percentile variant (HD95). A confidence interval (CI) was also computed throughout the models' training to assess the variability and noise in their performance. Gaussian behavior was tested through QQplot and subsequently the CI was derived using the 95% standard interval and it is reported as shown in Figure 2. Once the predictions were derived, we investigated the presence of statistical differences between models' prediction. The cranial caudal differences between the model predictions (UNet, SegResNetDS, DynUNet, and nnU-Net) and the clinician contours were quantitatively evaluated. As previously underlined, MedSAM2 was not evaluated for this, given the a priori bounding box. The cranial caudal differences were computed by comparing the first (cranial) and last (caudal) slices containing ground truth segmentation and predicted masks. The absolute difference in slice indices along the z-axis was taken. Then, the number of slices were properly converted in cm. The cranial caudal difference was then excluded, keeping the same value imposed for MedSAM2, in order to evaluate their volumetric performance. Before we tested the normality of our evaluation metrics, obtaining negative results. Therefore, we performed the non parametric Friedman test, to detect differences in metrics across the predictions of the six models on the same temporal test dataset. A *p*-value below 0.05 indicates a significant overall difference among the models. To identify which specific pairs of models differed significantly, we also conducted a *post-hoc* Conover test, based on the mean rank differences between models.

### 2.5 Probability Map derivation and residuals definition

The probability map was predicted for temporal test patients using the best models found in the previous section and, afterwards, CTV isoprobabilities were derived. The meaning of the 100% isoprobability region is a more conservative delineation (i.e., the intersection of all the models considered) compared to the full extent defined by the probabilistic map, while the lower percentage isoprobability region (voxels encompassed by at least one model) indicates broader contouring beyond the range of highest variability. They were then compared against the manual contours for each patient by using the following metrics: DCS, HD, HD95, ASD, VD. VD was set to be positive if the clinicians contoured larger volumens than the models (it is defined as clinical volume [cm^3^] - predicted isoprobability [cm^3^]).

To give an aggregate value to check the probability map goodness, the isoprobability contours were evaluated against the clinical CTV by quantifying two spatial components: (a) the portion of the clinical CTV not encompassed by the model's prediction at the 100% isoprobability threshold, called under-segmentation residual (Equation 1); (b) the region where the model's prediction at the lower percentage (lower %) isoprobability threshold extends outside the clinical CTV boundaries, called over-segmentation residual. These discrepancies were expressed as percentages of the CTV using the following relationships:


$$\begin{array}{l} \text{Under}-\text{segmentation residuals}\left(\%\right)=\frac{V_{100\%}-\left(V_{\text{clin}}\cap V_{100\%}\right)}{V_{\text{clin}}}\times 100 \end{array}$$ (1)

$$\begin{array}{l} \text{Over}-\text{segmentation residuals}\left(\%\right)=\frac{V_{\text{clin}}-\left(V_{\text{clin}}\cap V_{lower\%}\right)}{V_{\text{clin}}}\times 100 \end{array}$$ (2)

Together, these metrics characterize the proportion of the clinical CTV falling outside the 100%-lower% isoprobability confidence band.

## 3 Results

### 3.1 Times for training and prediction

Computational training times are reported in Table 2. All the models except MS-B reached convergence within 200 epochs with nnU-Net and MS-A exhibiting the lowest computational training time (≃5h). MS-B required almost three times longer to reach convergence, with a total of 500 training epochs. This behavior is likely due to the initialization strategy: MS-B was initialized with weights derived from a training process on different image types, whereas MS-A was initialized using weights obtained from training exclusively on CT images. Moreover, when the loss function was fixed, the computational time for SegResNetDS and DynUNet were doubled and tripled, respectively compared to UNet, which required 16 h (Table A1 in Appendix section). To compute predictions all the models employ less than 2 min per CT scan.

### 3.2 Training and hyperparameters optimization of UNet, SegResNetDS, and DynUNet

By exploring different hyperparameters with Optuna, optimal values were found for three out of six models considered. The hyperparameter search space is summarized in Table 3, where optimal values are in bold. Figure A1 in the Appendix shows the optimization process for these three models, including the DiceLoss function over epochs for different trials, some of which were pruned. The UNet model found with the HPO is the standard improved 3D UNet which uses 8–256 filters and strided convolutions to extract and downsample feature representations. In contrast, SegResNetDS adopts a specific encoder-decoder design with 16 initial filters and with deep supervision. This architectures increases computational cost and limits the number of feasible Optuna trials. DynUNet, with fewer tunable hyperparameters, exhibits similarly high computational demands as SegResNetDS. For this reason, the number of trials was also reduced for this model.

**Table 3:** Hyperparameters' range spaced for each of the three optimzed DL models.

Model	In-filter	Last-filter	Lr	Dp	Norm	Act Func
UNet	**8**, 16	**256**, 512	[1e-5, **1e-4**, 5e-3]	[0, **0.05**, 0.5]	batch, **instance**	prelu, **leaky**
SegResNetDS	8, **16**	perm (**2, 1, 4**, 2)	[**1e-5**, 5e-3]		batch, **instance**	prelu, **leaky**
DynUNet			[e-5, **0.001**, 5e-3]	[**0**, 0.5]	**batch**, instance	**prelu**, leaky

The best value found through Optuna is reported in bold. In columns are respectively: In and Last filter, Learning Rate (Lr), Dropout (Dp), Normalization (Norm), Activation Function (Act Func).

Once the optimization phase was completed, different loss functions were tested and the results are reported in the Appendix Table A1 (Dice scores on the validation dataset and computational times). As already observed during the optimization phase, training SegResNetDS and DynUNet required longer computational times, and this trend is preserved when using different loss functions. Also in this case, it can be observed that changing the loss function does not significantly affect the training time for each model, except for the HausdorffDT loss. In particular, the computational time for SegResNetDS and DynUNet was doubled and tripled, respectively, compared to UNet, which required 16 h (Table A1 in the Appendix section). Due to its slow convergence, the UNet model trained with the HausdorffDT loss was limited to 200 training images, as it required more than 30 h to converge. For this reason, the HausdorffDT loss was discarded for the remaining models.

Different metric performances on validation dataset during training are compared in Figure 2. UNet is smoother on validation dataset compared to DynUNet and it reaches higher performances compared to SegResNetDS.

### 3.3 Cranial caudal evaluation

**Figure 3:**
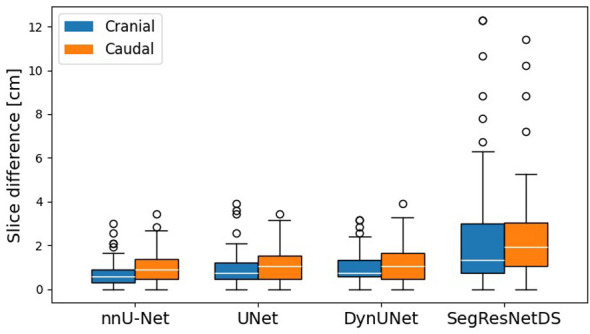
Cranial caudal differences. Difference in the sagittal plane of clinical and predicted contours in the caudal (orange) and cranial (blue) direction for all temporal test dataset. SegResNetDS predictions are the one with higher outliers number (points).

The cranial caudal differences were evaluated as described in Section 2.4, providing insight into how each model reproduces the superior-inferior extent of the CTV. In Figure 3, boxplots are presented showing the distribution of these differences in centimeters for each model on temporal test cohort. SegResNetDS exhibits the highest variability, indicating less consistent agreement with clinician contours along the cranial caudal axis, while UNet, DynUNet, and nnU-Net show more compact distributions. Cranial difference is smaller than caudal difference on average and also for what regards the standard deviation for all the four models shown (Figure 3), with median cranial difference of 0.6, 0.75, 0.75, and 1.35 cm, respectively for nnU-Net, UNet, SegResNetDS, and DynUNet.

### 3.4 DL comparison on temporal test dataset

**Figure 4:**
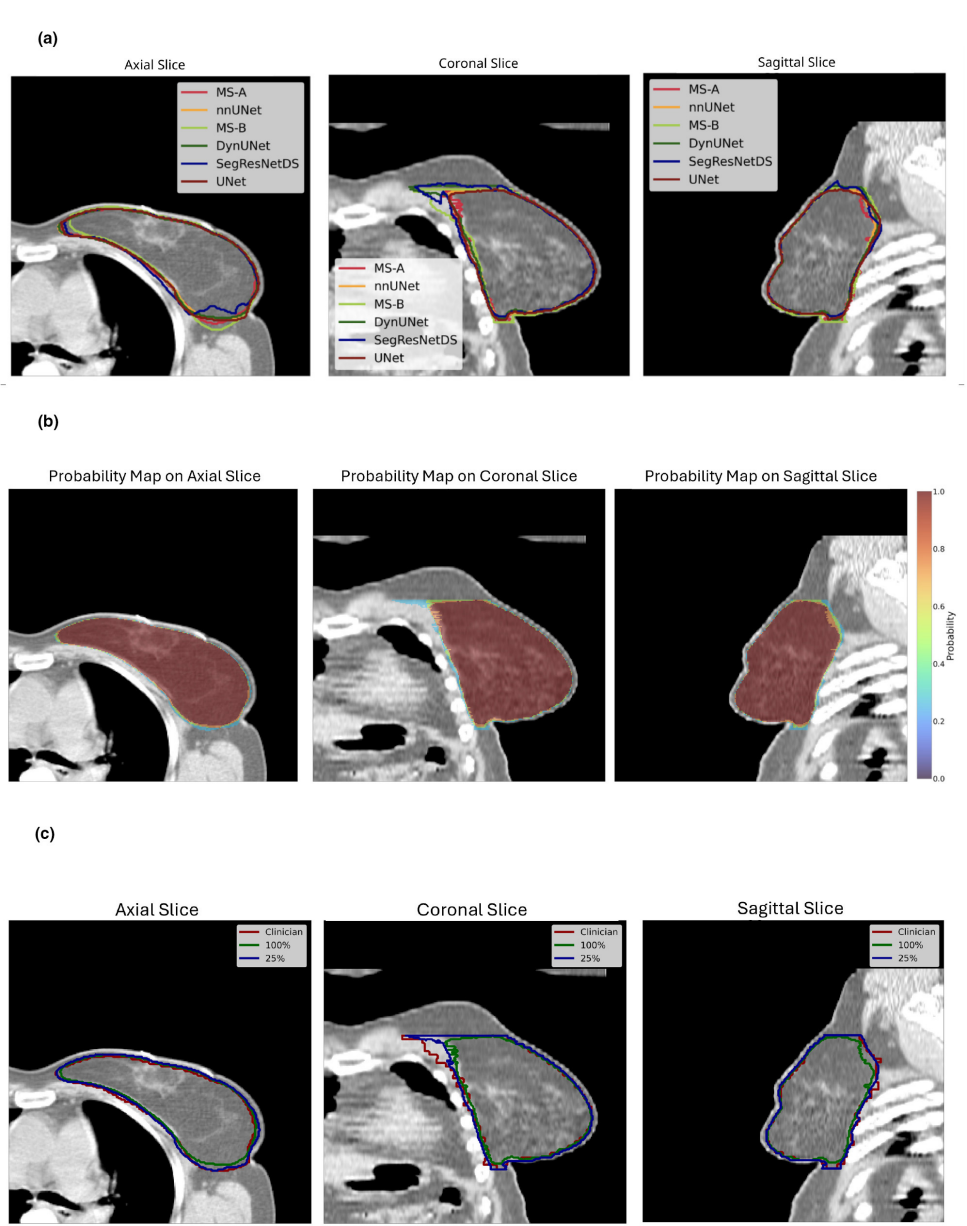
Left breast CTV example across three planes. **(a)** Six model predictions comparison without cranial caudal cropping. MS-A is MedSAM2-A, MS-B is MedSAM2-B. **(b)** Probabilistic map (rainbow colormap) derived from cranial caudal cropped predictions of the four best performing models (UNet, DynUNet, nnU-Net, and MedSAM2-A) shown on top of the corresponding CT. **(c)** Isoprobability contours derived from the probabilistic map with cranial caudal cropping applied. Clinical CTV (red) compared with model-derived isoprobability contours at 25% (blue) and 100% (green).

**Figure 5:**
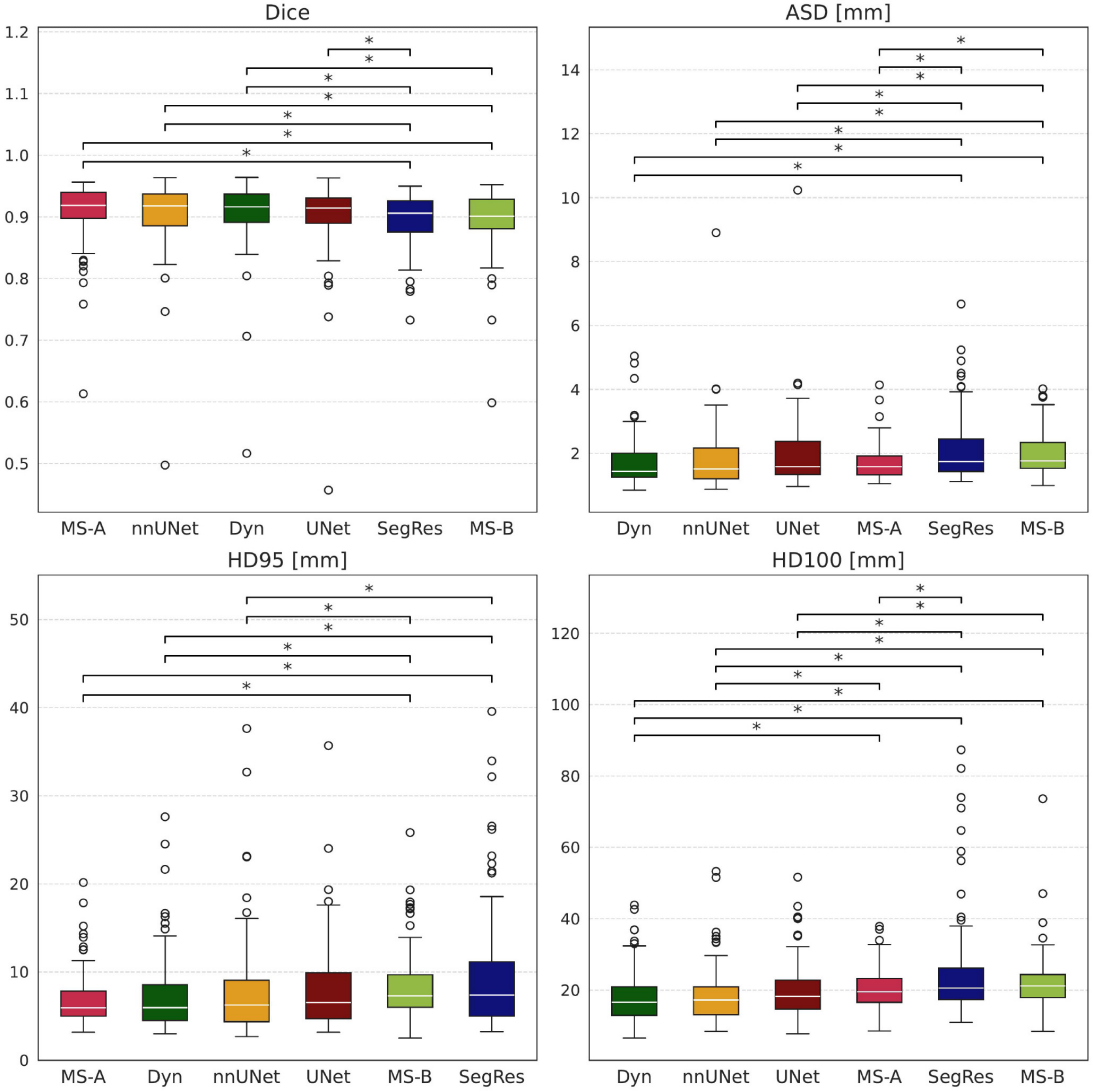
Model performance comparison with cranial-caudal cropping on temporal test dataset. Boxplots show Dice, Average Surface Distance (ASD), Hausdorff distance, and its 95th percentile for UNet (red), SegResNetDS (blue), DynUNet (darkgreen), nnU-Net (yellow), MS-A (pink) and MS-B (light green). Boxplots are ordered by median (white line): descending for metrics optimal at 1, ascending for metrics optimal at 0. Plots are zoomed excluding three SegResNetDS outliers for clarity in visualization. Asterisks and lines on top images indicate significant differences via Conover test.

Once the best models were found, CTV predictions on the temporal test dataset were generated (left side patient example in Figure 4a, righ side patient example in Appendix Figure A4a), and the following metrics were computed to evaluate model performance: Dice, IoU, ASD, HD, HD95, and VD. To ensure a fair comparison across models, the same built-in cropping strategy (generation of a bounding box a priori) used by MedSAM2 was applied uniformly (Section 2.3). This approach yielded segmentations that did not differ along the cranial caudal axis compared with the clinician provided segmentations. In Figure 5 consequently, the original differences in cranial and caudal directions (reported in Figure 3) disappeared. The mean and standard deviations values for each metric and each model are reported in Table 4, while the boxplots are shown in Figure 5, with models in decreasing ordered with their mean distribution. The Friedman test indicated global significance for all metrics, and the *p*-values from the subsequent Conover tests are reported in Table 5. In bold are represented *p*-values < 0.05 that show statistical difference between the models. Across all metrics, UNet, DynUNet, nnU-Net, and MS-A achieved comparable performance, with no statistically significant differences observed among these models. These models consistently obtained high overlap scores (Dice ≥ 0.91) and low surface distance values. In contrast, SegResNetDS and MS-B showed significantly different performance compared to the remaining models in nearly all metrics. Starting from a model that has already been fine-tuned on CT (such as MS-A) allows achieving good results with significantly fewer epochs, whereas an overly generic model (such as MS-B) does not automatically guarantee better performance. Moreover, SegResNetDS has a visibly higher number of outliers in distribution plots (that were omitted in Figure 5). In contrast, UNet, DynUNet, nnU-Net and MS-A did not exhibit statistically significant differences among each other, suggesting comparable performances across the evaluated cohort (Table 5).

**Table 4:** Mean and standard deviation metric values derived on temporal test dataset for the six models trained.

Model	Dice	IoU	HD95 (mm)	HD100 (mm)	ASD (mm)	VD (*cm*^3^)
UNet	0.91 ± 0.06	0.84 ± 0.08	6.50 ± 5.00	18.00 ± 8.00	1.58 ± 1.10	0 ± 70
SegRes	0.90 ± 0.10	0.82 ± 0.10	8 ± 20	21 ± 24	2 ± 12	−11 ± 100
Dyn	0.92 ± 0.06	0.85 ± 0.08	5.90 ± 4.50	**16.00** **±7.00**	**1.44** **±0.79**	12 ± 66
nnU-Net	**0.92** **±0.06**	**0.85** **±0.08**	6.20 ± 5.60	17.00 ± 8.00	1.50 ± 1.00	−7 ± 70
MS-A	**0.92** **±0.05**	**0.85** **±0.07**	**5.90** **±3.00**	19.60 ± 5.80	1.58 ± 0.54	24 ± 60
MS-B	0.90 ± 0.05	0.82 ± 0.07	7.30 ± 3.90	21.20 ± 7.70	1.70 ± 0.70	36 ± 70

Metrics reported are: Dice, IoU, ASD, HD95 HD100 and Volume Difference (VD) computed as (Predicted-Ground Truth volume). Best values (per metric) are in bold. Metrics were computed on predictions with cranial-caudal crop applied.

**Table 5:** Results of the *post-hoc* Conover test performed on the distributions of segmentation metrics obtained from six DL models.

	MS-A	MS-B	SegRes	UNet	nnU-Net
Dice					
Dyn	7.3e-01	**1.6e-02**	**2.5e-03**	4.3e-01	9.6e-01
MS-A		**6.0e-03**	**7.6e-04**	2.5e-01	7.6e-01
MS-B			5.3e-01	1.1e-01	**1.4e-02**
SegRes				**2.6e-02**	**2.2e-03**
UNet					4.0e-01
ASD					
Dyn	4.8e-01	**9.0e-05**	**5.3e-05**	5.2e-02	5.8e-01
MS-A		**1.2e-03**	**7.9e-04**	2.1e-01	8.8e-01
MS-B			9.0e-01	**4.6e-02**	**7.4e-04**
SegRes				**3.4e-02**	**4.6e-04**
UNet					1.6e-01
HD95					
Dyn	9.4e-01	**9.8e-04**	**4.4e-04**	1.0e-01	5.7e-01
MS-A		**1.3e-03**	**5.9e-04**	1.2e-01	6.3e-01
MS-B			8.2e-01	9.4e-02	**6.2e-03**
SegRes				5.8e-02	**3.1e-03**
UNet					2.8e-01
HD100					
Dyn	**1.3e-03**	**4.3e-07**	**1.3e-07**	5.5e-02	8.2e-01
MS-A		5.9e-02	**3.5e-02**	1.9e-01	**2.8e-03**
MS-B			8.2e-01	**1.5e-03**	**1.3e-06**
SegRes				**6.6e-04**	**4.2e-07**
UNet					9.1e-02

Values in bold indicate statistically significant pairwise differences (*p* < 0.05).

### 3.5 Probability map and segmentation residuals

**Figure 6:**
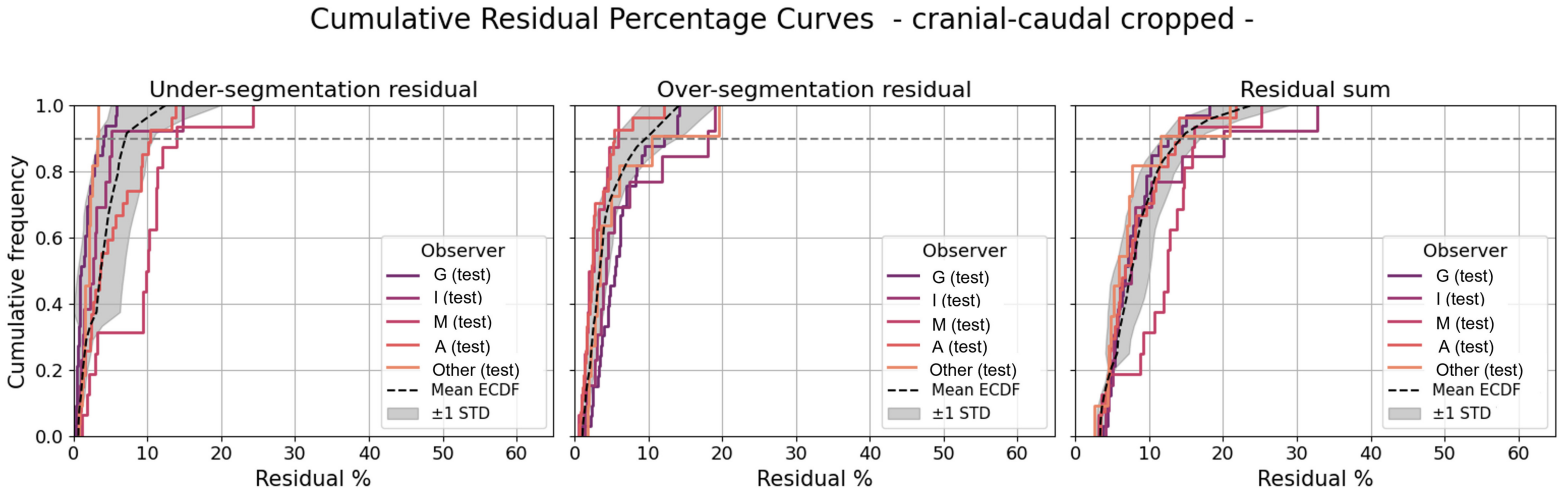
Cumulative residuals over clinical contours for labels with cranial caudal crop. Gray line and band refer to test dataset (average and std of the cumulative clinical frequency), while G, I, M and A refer to the different clinicians. Horizonal line at 0.9 is the set threshold to check the cumulative frequency.

Four models were found to be the best ones and comparable as previously underlined: UNet, DynUNet, nnU-Net, and MS-A. By averaging their boolean predictions, the probability map was derived (see Figure 4b for a left-side patient and Figure A4B in Appendix for a right-side patient): considering that four are the models, four are the isoprobabilities consequently derived (25%, 50%, 75%, 100%). Examples of the 25 and 100% isoprobability segmentations, compared with clinician provided contours, are shown in Figure 4c for a left-side patient and in Appendix Figure A4c for a right-side patient. In Equation 2 *V*_*lower%*_ is replaced to *V*_25%_. As described in Section 2.5, the Over and Under segmentation residual were computed by using predictions with cranial caudal crop applied (Figure 6). The Residual sum (their combination) was also shown. The cumulative frequency of 0.9 (black dashed line in Figure 6) is reached at : 6.9% ± 3.7 for under segmentation residuals, 9.6% ± 4.1 for over segmentation residuals, 14.2%± 2.5 for residual sum. Finally, difference in 3D volume between 100 and 25% isoprobabilities computed for all 100 patients post 2021 are on average of 123 cm^3^, with a median of 100 cm^3^ and a standard deviation of 81 cm^3^.

## 4 Discussion

This study compared the performance of six state-of-the-art neural networks for clinical CTV segmentation in whole-breast radiotherapy. Three MONAI-based models were optimized using Optuna and trained for 200 epochs, while the remaining three models (nnU-Net, MedSAM2-A, and MedSAM2-B) were only trained and temporally validated without extensive hyperparameter optimization. MedSAM2 required an integration with clinician input to make the prediction: a spatial prompt, as underlined in their descriptive paper (Ma et al., 2025), not needed from the others. All the remaining four models required an automatic ad hoc post-processing to enhance performance. For UNet, SegResNetDS, DynUNet, and nnU-Net, the applied post-processing, which simply removes smaller disconnected volumes while preserving the largest one, can be easily provided to clinicians without additional effort. This model comparison and HPO strengthen the previous result by Ubeira-Gabellini et al. (2025). The parameters of the UNet model described are confirmed to be the best ones after HPO with Optuna. Moreover, the model trained over the same large cohort of patients (*N* = 861), larger than previously analyzed (Buelens et al., 2022; Baroudi et al., 2023), was found to be stable over time. The temporal split was chosen to simulate a real-world deployment scenario, where models trained on previous cases are applied to subsequently treated patients, rather than to explicitly assess long-term model degradation or dataset shift. While the proposed model was evaluated using conventional geometric metrics, it is acknowledged that a more comprehensive assessment could include qualitative expert review and dosimetric analysis of the resulting treatment plans (Mazonakis et al., 2025; Poel et al., 2025). Such evaluation is left for future work.

This work enlarges the finding of our previous work (Ubeira-Gabellini et al., 2025), focused on the UNet model, by adding other five models. All the models trained and tested operated in 3D (except MedSAM2) and are capable of simultaneously processing both right and left CTV breast regions, differently from previous literature. The predicted segmentations of all models closely match clinician contours after careful pre- and post-processing, HPO and/or model fine tuning. When comparing other considered models to UNet results, the trend of smaller differences near the skin and chest sides is confirmed, while greater uncertainty is observed laterally and at the cranial-caudal borders (see Figure 3), reflecting areas of higher inter-observer variability. In particular SegResNetDS, has the highest cranial caudal differences with outliers up to 10 cm. To make fair the volumetric comparison, considering the a priori bounding box of MedSAM2, a cranial caudal crop was applied to all predictions. In particular, the *Post-hoc* Conover (Table 5) analysis, evaluating significant *p*-values in pairwise comparisons, revealed that SegResNetDS and MedSAM2-B underperform compared to UNet, DynUNet, nnU-Net, and MS-A, with *p*-values below or approximately below 0.05 for Dice, ASD, HD95, and HD100. Moreover, the average of SegResNetDS and MS-B is lower for all metrics, and distributions have larger standard deviations, see Table 4. The other four models, instead, reached high accuracy on all metrics (Dice≃0.91, IoU≃0.84, ASD≃1.5 mm) and did not show significant differences among them: their segmentations are comparable and consistent, with variability mostly within expected IOV (Maddaloni et al., 2025). Although MedSAM2-A achieved promising results - showing the best mean values in the temporal test dataset for almost all metrics - its performance remains dependent on the availability of an accurate a priori bounding box. As MedSAM2 requires a bounding box prompt to generate predictions, we evaluated possible alternatives, including clinician-drawn boxes, omission of the prompt, or model-generated boxes. An addition clinical *a priori* bounding box was impractical and subject to IOV; omitting the prompt may lead to suboptimal performance; and using another model could introduce bias. We therefore employed ground-truth–derived bounding boxes to approximate clinical workflow, explicitly acknowledging this as a methodological limitation for potential data leakage. Moreover, considering the execution time, SegResNetDS and DynUNet required longer training times (≃36 h and ≃49 h, respectively), with nnU-Net and MS-A showing the best timinig performance (≃5 h). Most studies do not perform a systematic comparison across multiple architectures (e.g., stacking, ensemble approaches). In addition, they typically focus just on UNet and its variants (such as V-Net or SegResNet), without exploring more advanced models like MedSAM2 or open-source solutions such as nnU-Net from TotalSegmentator. Just few are the studies comparing DL models for breast CTV segmentation on CT; Hou et al. (2023) considered, different models and lower numbers, obtaining similar performances without any significant differences in terms of Dice between the model architectures. The same work underlined how performances are highly influenced by the IOV in the manual delineation, which is dependent on the clinical context and medical expertise. Few other comparing models' study were performed, neglecting the CTV (Narimani et al., 2025; Erdur et al., 2024). Seldom, comparisons are limited to custom-trained vs. vendor-pretrained models (Chen et al., 2024) or between different commercial solutions (Hou et al., 2023). Another study estimated uncertainty in CTV delineation in CBCT images with attention-based semi-supervised models (Wang et al., 2024).

Finally, the probability map was derived by combining the predictions of the four best-performing models, providing a robust measure of segmentation model confidence. Such ensemble-derived probabilistic maps capture consensus information across models, highlight regions of higher and lower confidence, and an support more informed decision-making in treatment planning by visualizing areas of uncertainty rather than ignoring them. To our knowledge, this represents one of the first applications of ensemble-based agreement visualization in the context of breast CTV segmentation within radiotherapy planning. Analysis of residuals at a cumulative frequency of 0.9 shows that under- and over-segmentation deviations remain moderate (≃7%–10% for under- and over-segmentation, 14% for the residual sum), indicating good agreement of personalized probability map based on best performing models with clinician contours. The average 3D volume difference between the 100 and 25% isoprobability contours (123 cm^3^) highlights regions of higher uncertainty, particularly at the CTV cranial caudal borders, consistent with areas of higher inter-observer variability. These results suggest that the probability map could guide clinician review, helping focus attention on regions of lower confidence and potentially improving both accuracy and efficiency in contour verification.

Overall, the comparable performances across models suggest that the consistency, quality, and quantity of annotated data is expected to have a greater impact on model performance than the specific DL architecture employed. In particular, it appears difficult to surpass the inherent inter-observer variability (IOV) of the CTV contouring process (Maddaloni et al., 2025). Finally, deep ensemble methods used to derive uncertainty maps have not yet been explored for CTV segmentation in breast radiotherapy on CT, despite their proven utility in diagnostic imaging, being this the first in the field: ensemble and uncertainty approaches have been explored in diagnostic imaging [ultrasound [e.g., Musah et al., 2025; Islam et al., 2024, histopathology Schmidt-Santiago et al., 2025), but not for auto-contouring of breast CTV. Our suggested approach would give an estimate of an uncertainty in CTV estimate due to the type of model used, explicitly showing the impact of the intrinsic uncertainties in AI-based segmentation. And this would make the clinicians more aware of the uncertainty of “black-box” tools: surely, further research in this field is warranted.

## Data Availability

The data analyzed in this study is subject to the following licenses/restrictions: the dataset consists of retrospective, anonymized CT scans and is not publicly available. Access is restricted to authorized researchers under institutional and ethical approval. Requests to access these datasets should be directed to Unità Operativa di Radioterapia, Ospedale San Raffaele, radioterapia@hsr.it.
