# Supplementary material for: Deep ensemble optimized models for probabilistic CTV breast segmentation
Source: Front Artif Intell. 2026 Apr 30;9:1777653. doi: 10.3389/frai.2026.1777653 (PMC13171566; doi:10.3389/frai.2026.1777653)
Supplement: Supplementary file 1 [file Supplementary_file_1.pdf]

frontiers

Deep ensemble optimized models for probabilistic CTV breast segmentation

Cecilia Riani[†,1,2]**, Maria Giulia Ubeira-Gabellini**[†,1,*]**, Gabriele Palazzo**[1]**, Giuseppe Ricciardi**[1]**, Antonella del Vecchio**[1]**, Alessandra Palma**[3]**, Anna Balsamo**[4]**, Angela Coniglio**[4] **and Claudio Fiorino**[1]

[1] *Medical Physics, IRCCS San Raffaele Scientific Institute, Milan, Italy.*
[2] *Radiation Biophysics and Radiobiology Laboratory, Physics Department, University of Pavia, Pavia, Italy.*
[3] *Centro Nazionale Intelligenza Artificiale e Tecnologie Innovative per la Salute, Istituto Superiore di Sanità, Rome, Italy.*
[4] *Ministry of Health, Department of Human Health, Animal Health and Ecosystem (One Health) and International Relations (DOHRI), Rome, Italy.*
† *These authors contributed equally to this work and share first authorship.*

Correspondence*:
Maria Giulia Ubeira-Gabellini
ubeira.mariagiulia@hsr.it

1 SUPPLEMENTARY MATERIAL

1.1 HPO and fine tuning

The HPO was performed through Optuna for UNet, SegResNetDS and DynUNet architectures. The function minimized was the DiceLoss function. Fig. A1 reports the optimization process for the three models. The pruner method allowed to discard non-minimum hyperparameters combinations to the first epochs. Once the HPO was performed the different loss functions were also attempted to understand with which one the models attained convergence faster and smoothly (Tab. A1). Fig. A2 show the loss varying with epochs. The DiceLoss was employed for UNet and SegResNetDS; while DiceCEloss was used for DynUNet and Soft Dice Loss plus Cross Entropy Loss was employed for nnUNet. All models predictions were subsequently post-processed with the cropping procedure explained in [1]. SegResNetDS was the models the most subjected to this cropping Fig. A3.

**Training Loss**	Dice		DiceCE		Tversky		HausdorfDT	
	Val Dice	time	Val Dice	time	Val Dice	time	Val Dice	time
UNet	**0.90**	**16h**	0.89	16h	0.89	16h	0.74	32h*
SegResNetDS	**0.88**	**36h**	0.88	37h	0.86	36h	/	/
DynUNet	0.90	49hr	**0.90**	49h	0.90	49hr	/	/

Table A1. Train Loss function. Dice validation values for Unet, SegResNetDS and DynUNet trained with three/four different loss function alternatively. The corresponding time for model training with all images for 200 epoch are also reported. The asterisk (*) refers to model trained with only 200 images, instead of the full dataset. In bold the best train loss per each models in terms of metric, time and smoothness to reach convergence.

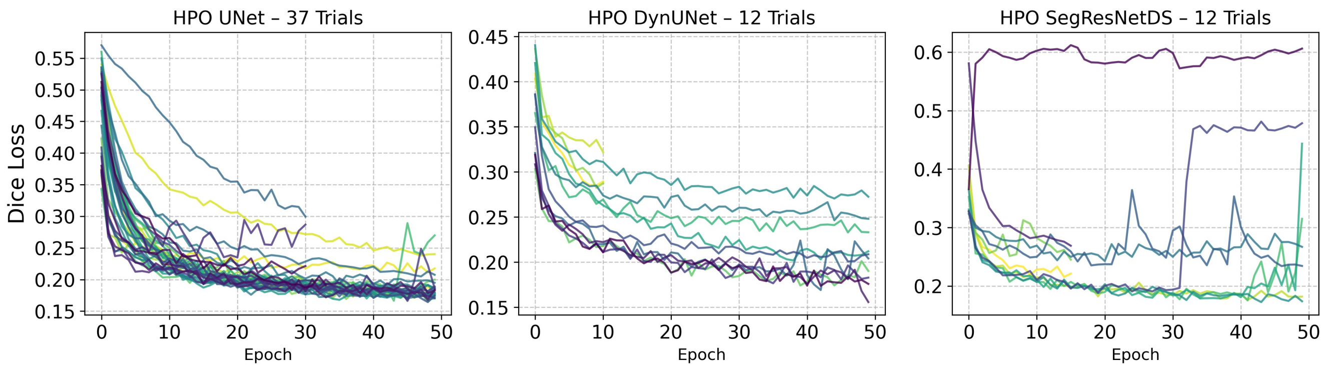

Figure A1. Hyperparameters optimization process through Optuna for the three architectures. From the left UNet, SegResNetDS and DynUNet. Each color (a specific trial) represents the training DiceLoss over 50 epochs with a specific set of hyperparamenters.

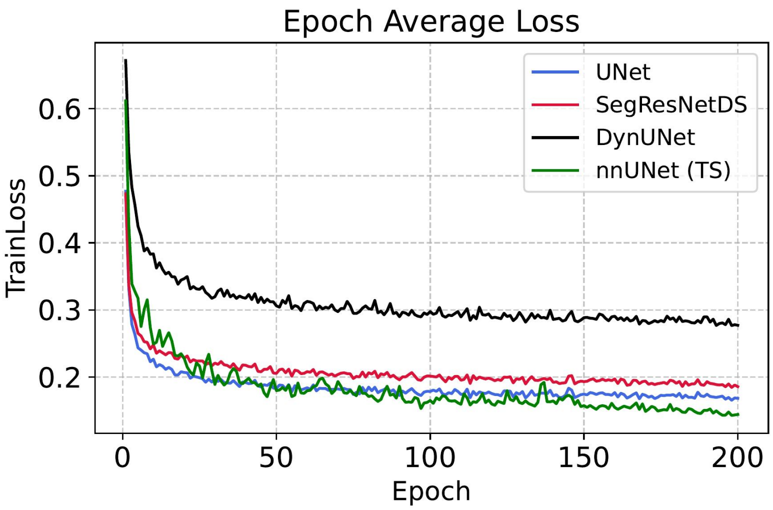

Figure A2. TrainLoss comparison. DiceLoss for UNet and SegResNetDS; DiceCEloss for DynUNet and Soft Dice Loss plus Cross Entropy Loss for nnUNet.

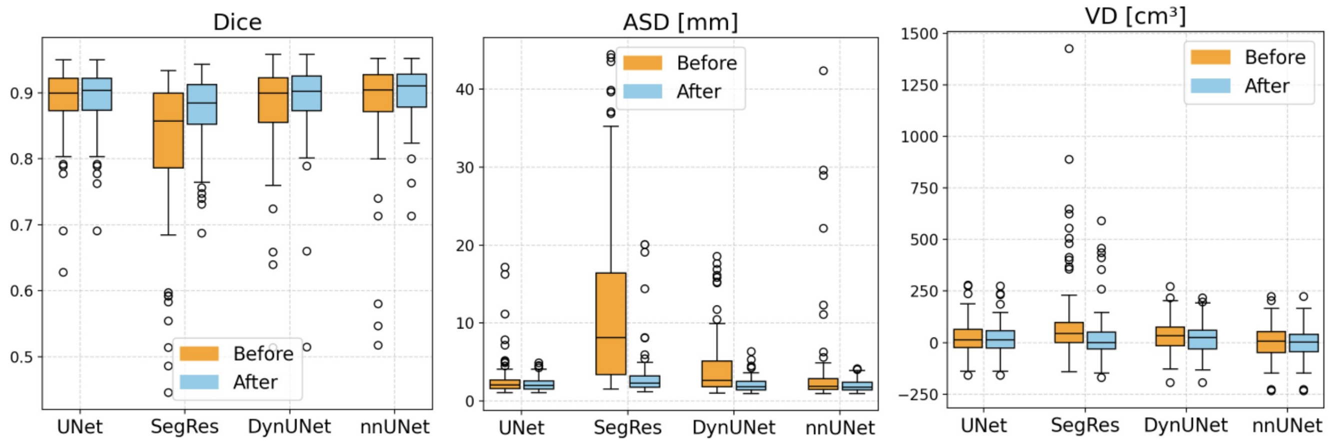

Figure A3. Boxplots of Metrics prediction before and after post-processing. In blue the prediction after post-processing (detailed in [1]), in orange the prediction before post-processing.

1.2 Best Model ensembling

Figures A4a-A4c show the same results as in Section 3, but for the right side CTV patient: Fig. A4a shows the six segmentations model comparison, Fig. A4b shows the probability map derived from the four best performing models and Fig. A4c shows the comparison between the two isoprobabilities 100% and 25% and the ground truth clinician segmentation. Supplementary boxplots illustrating the per-metric distribution of segmentation performance for the best evaluated models are reported in Figure A5. Results refer to the full test cohort of 100 patients. The ensemble strategy consistently achieves comparable performance across all metrics.

1.3 Comparison with MIM software

The best UNet model found after HPO was compared with MIM software latest version 2024 (Fig. A6). Our built in model attained better median performances on all metrics.

1.4 Inter-observer variability

Finally, it was analyzed the UNet performance back on each different clinical contours (see Tab. 1), obtaining different performances in terms of DSC and VD as shown in Fig. A7. In particular, Clinician I - the one with highest numbers - achieves better performance, likely due to a greater influence on the training dataset. These results suggest that developing ad-hoc models tailored to each clinician could increase metric performance. However, such an approach would move towards more individualized procedures rather than promoting a standardized, common methodology, as this work has done.

REFERENCES

[1]Maria Giulia Ubeira-Gabellini, Gabriele Palazzo, Martina Mori, Alessia Tudda, Luciano Rivetti, Elisabetta Cagni, Roberta Castriconi, Valeria Landoni, Eugenia Moretti, Aldo Mazzilli, Caterina Oliviero, Lorenzo Placidi, Giulia Rambaldi Guidasci, Cecilia Riani, Andrei Fodor, Nadia Gisella Di Muzio, Robert Jeraj, Antonella del Vecchio, and Claudio Fiorino. Development and external multicentric validation of a deep learning-based clinical target volume segmentation model for whole-breast radiotherapy. *Physics and Imaging in Radiation Oncology*, 34:100749, April 2025.

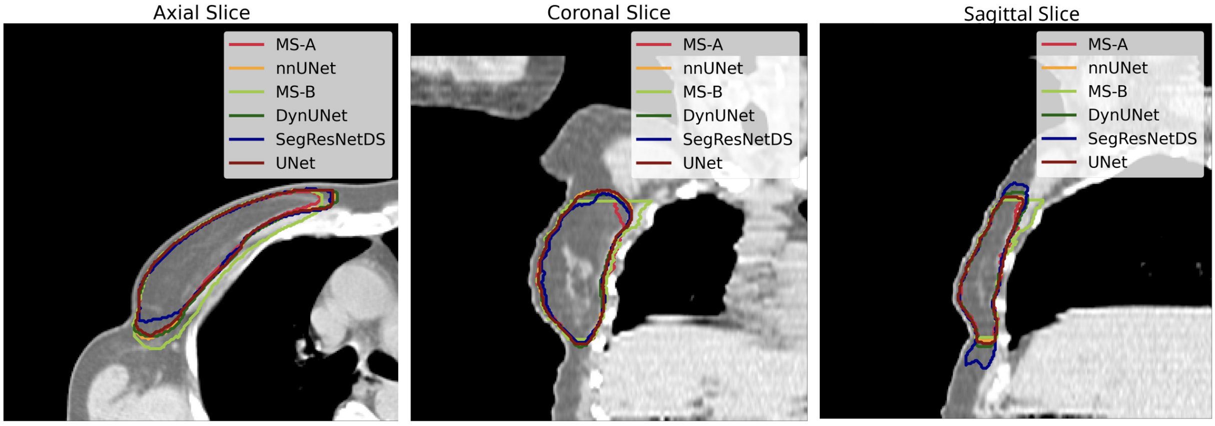

Figure A4a. Six model predictions comparison without cranial caudal cropping. MS-A is MedSAM2-A, MS-B is MedSAM2-B.

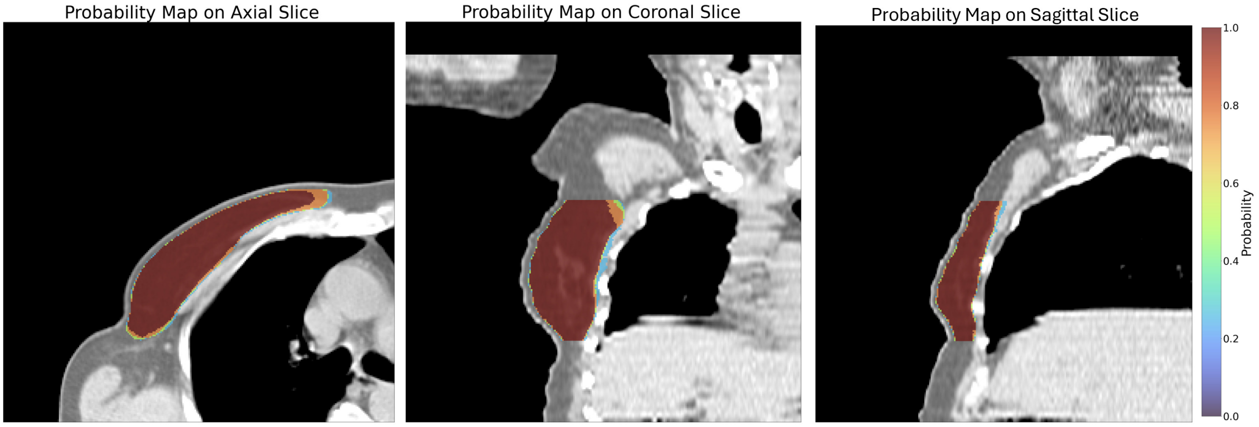

Figure A4b. Probabilistic map (rainbow colormap) derived from cranial caudal cropped predictions of the four best performing models (UNet, DynUNet, nnU-Net, and MedSAM2-A) shown on top of the corresponding CT.

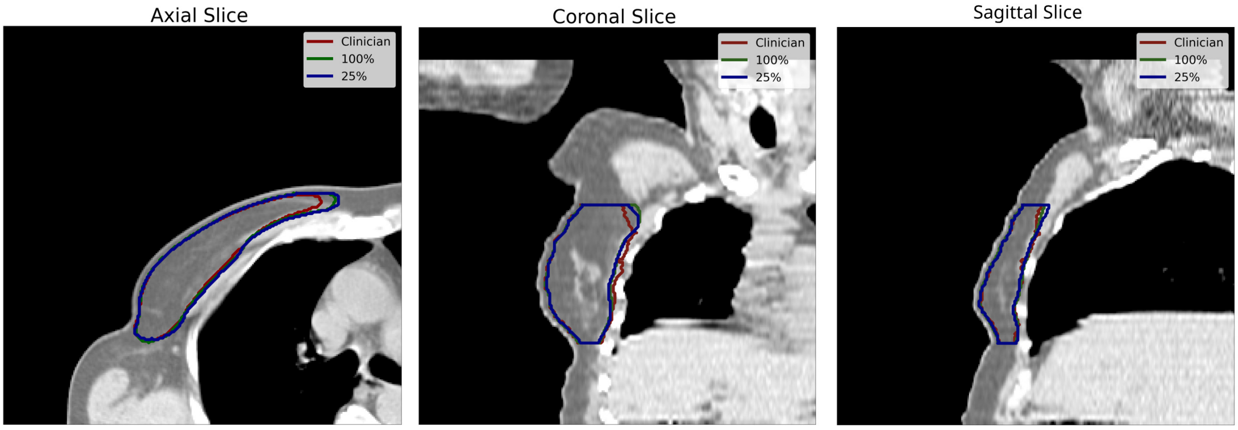

Figure A4c. Isoprobability contours derived from the probabilistic map with cranial caudal cropping applied. Clinical CTV (red) compared with model-derived isoprobability contours at 25% (blue) and 100% (green).

Figure A4. Right breast CTV example across three planes.

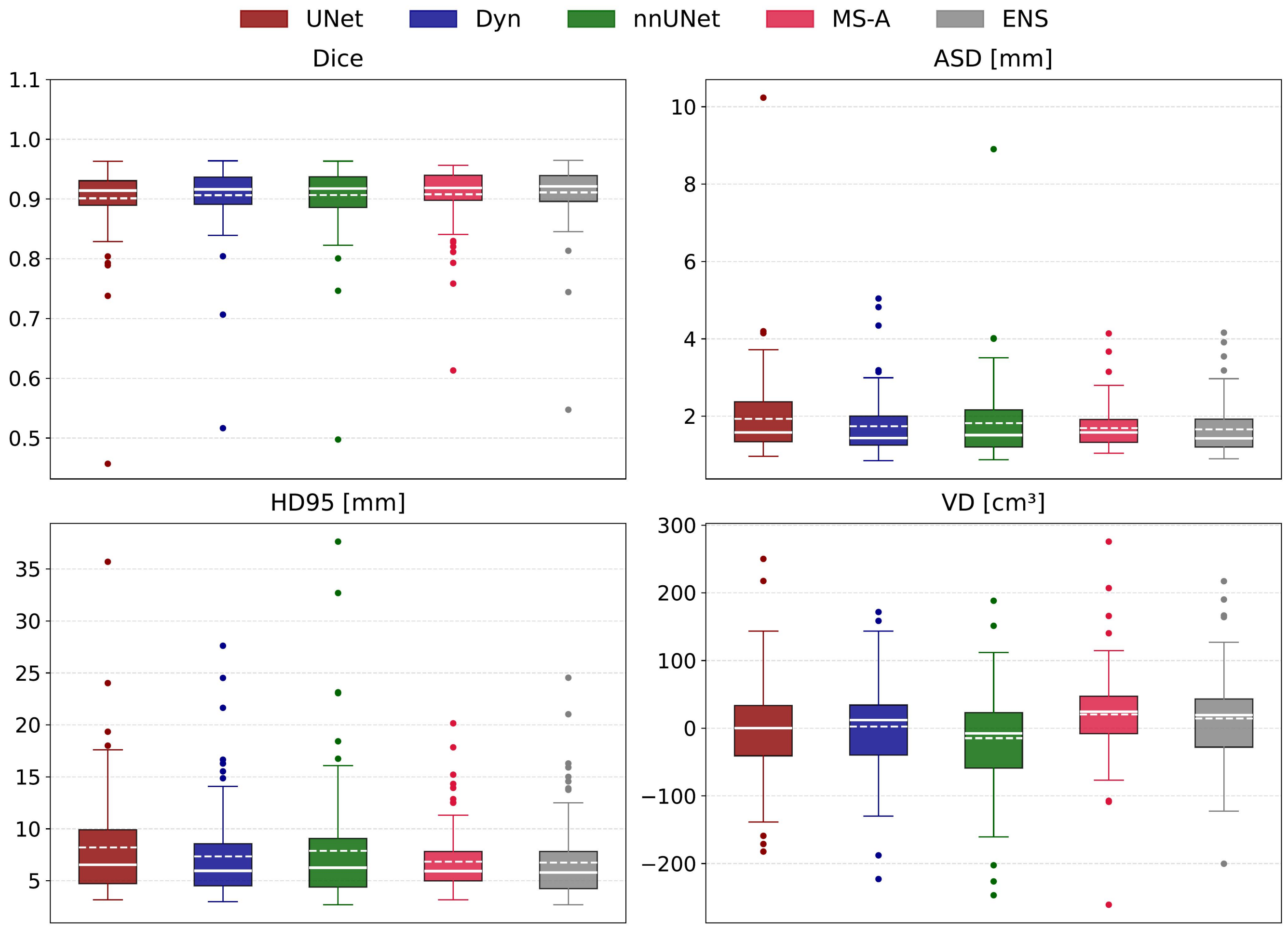

Figure A5. Comparison of segmentation performance across the four best models (UNet, MS-A, nnUNet, DynUNet) and their ensemble (ENS) on temporal test cohort of 100 patients. Boxplots display the distribution of Dice, ASD, HD95, and VD for each model. The horizontal line and dashed line within each box represent the median and mean, respectively.

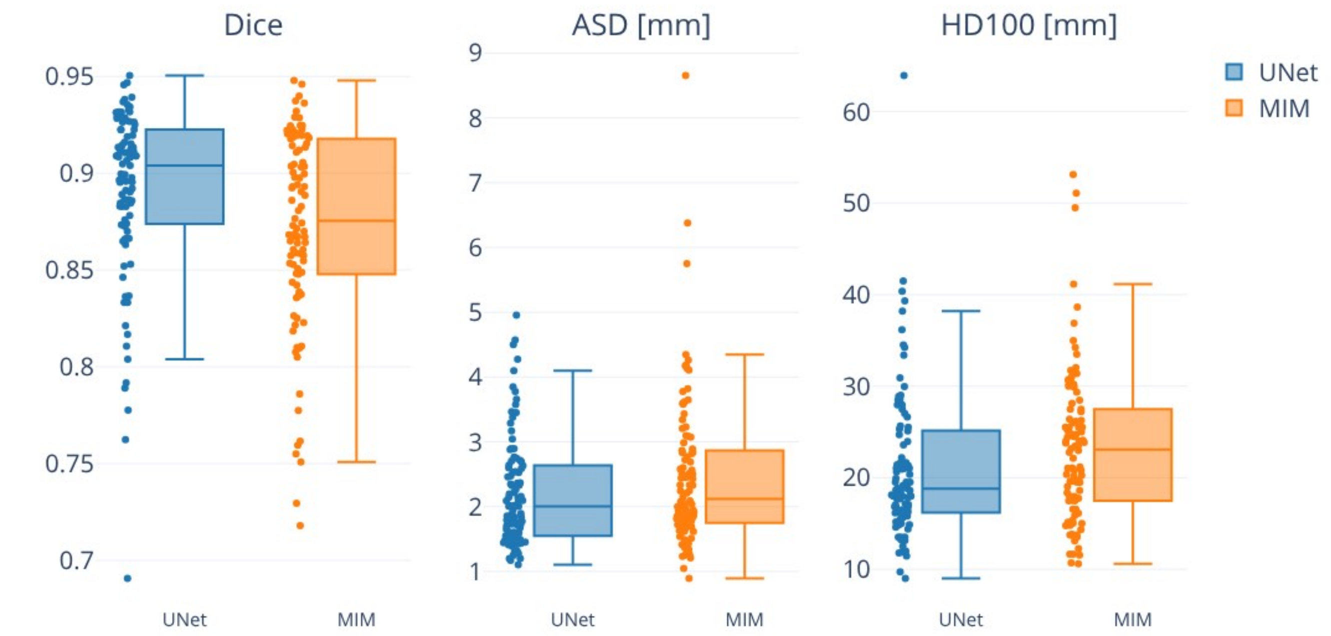

Figure A6. Boxplots of Metrics prediction for UNet and MIM model. In blue UNet predictions, in orange MIM software predictions.

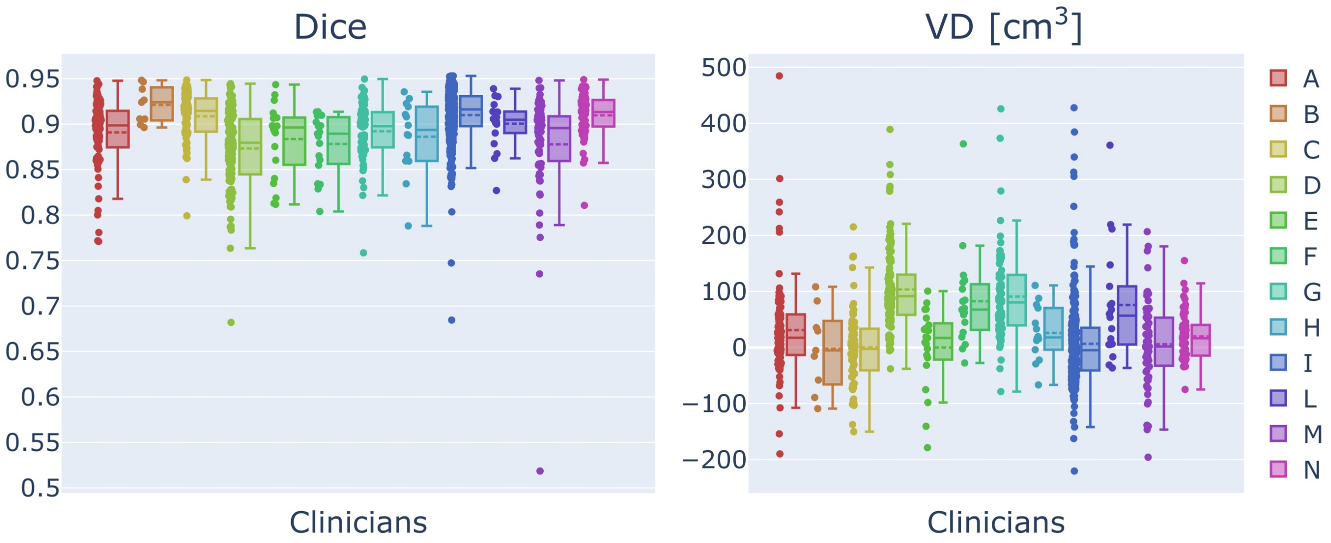

Figure A7. Model performance over different clinicians. The figure shows diverse Dice and Volume difference (VD) boxplots computed on UNet predicted values against the labels produced by different clinicians. The twelve letters [A-N] represents the twelve different clinicians who segmented the images used for model's train and validation. In this figure, predictions of O and unkonwn (see Tab. 1) were excluded because of unknown referring clinician or low numbers. Only data before 2022 was considered.
